# Expression Quantitative Trait Loci (eQTL) Mapping in Puerto Rican Children

**DOI:** 10.1371/journal.pone.0122464

**Published:** 2015-03-27

**Authors:** Wei Chen, John M. Brehm, Jerome Lin, Ting Wang, Erick Forno, Edna Acosta-Pérez, Nadia Boutaoui, Glorisa Canino, Juan C. Celedón

**Affiliations:** 1 Division of Pulmonary Medicine, Allergy and Immunology, Children’s Hospital of Pittsburgh of the University of Pittsburgh Medical Center, Pittsburgh, Pennsylvania, United States of America; 2 Department of Human Genetics, Graduate School of Public Health, University of Pittsburgh, Pittsburgh, Pennsylvania, United States of America; 3 Behavioral Sciences Research Institute, University of Puerto Rico, San Juan, Puerto Rico; Harvard Medical School, UNITED STATES

## Abstract

**Background:**

Expression quantitative trait loci (eQTL) have been identified using tissue or cell samples from diverse human populations, thus enhancing our understanding of regulation of gene expression. However, few studies have attempted to identify eQTL in racially admixed populations such as Hispanics.

**Methods:**

We performed a systematic eQTL study to identify regulatory variants of gene expression in whole blood from 121 Puerto Rican children with (n = 63) and without (n = 58) asthma. Genome-wide genotyping was conducted using the Illumina Omni2.5M Bead Chip, and gene expression was assessed using the Illumina HT-12 microarray. After completing quality control, we performed a pair-wise genome analysis of ~15 K transcripts and ~1.3 M SNPs for both local and distal effects. This analysis was conducted under a regression framework adjusting for age, gender and principal components derived from both genotypic and mRNA data. We used a false discovery rate (FDR) approach to identify significant eQTL signals, which were next compared to top eQTL signals from existing eQTL databases. We then performed a pathway analysis for our top genes.

**Results:**

We identified 36,720 local pairs in 3,391 unique genes and 1,851 distal pairs in 446 unique genes at FDR <0.05, corresponding to unadjusted P values lower than 1.5x10^-4^ and 4.5x10^-9^, respectively. A significant proportion of genes identified in our study overlapped with those identified in previous studies. We also found an enrichment of disease-related genes in our eQTL list.

**Conclusions:**

We present results from the first eQTL study in Puerto Rican children, who are members of a unique Hispanic cohort disproportionately affected with asthma, prematurity, obesity and other common diseases. Our study confirmed eQTL signals identified in other ethnic groups, while also detecting additional eQTLs unique to our study population. The identified eQTLs will help prioritize findings from future genome-wide association studies in Puerto Ricans.

## Introduction

Identification of sources of variability in gene expression is a critical step in our understanding of biological mechanisms of common diseases such as asthma. In humans, transcript abundance is largely regulated by genetic variants.

Expression quantitative trait loci (eQTL) are defined as genomic loci that regulate expression of mRNA. eQTLs provide insight into gene networks and disease pathogenesis, and help enhance and interpret results from genome-wide association studies (GWASs). Concurrent data on genome-wide (GW) genotypes and mRNA allow us to identify eQTLs, ultimately enhancing our interpretation of GWASs of complex diseases by helping identify functional variants [1–3]. On the basis of the physical distance between a SNP and a transcript, there are two types of eQTL effects: local and distal, which are alternatively called cis and trans. A local effect is identified when a regulatory SNP is within certain genomic distance (e.g. 1Mb) of the transcript starting site (TSS), and implies a direct regulatory effect of the SNP. In contrast, a distal effect is identified with a regulatory SNP that is much further from a TSS, or on a different chromosome, which implies that the eQTL acts through an intermediary transcription factor. To date, few eQTL analyses have been conducted in Hispanic subgroups, and no such study has been reported in Puerto Ricans.

Puerto Ricans are a racially admixed population, with one study reporting average European, African and Native American ancestries of 64%, 24%, and 12%, respectively [4]. For reasons that are unclear but likely to include genetic susceptibility, Puerto Ricans are disproportionately affected with diseases such as obesity, prematurity and asthma [5, 6]. Understanding eQTL patterns in Puerto Ricans should help us elucidate both regulation of gene expression and disease causation in racially admixed populations in general, and in Hispanic subgroups in particular.

In this report, we present a comprehensive survey of genome-wide eQTL mapping using both GW genotypic data and GW whole-blood mRNA in 121 Puerto Rican children who participated in a study of asthma. We thus deliver the first eQTL database in Puerto Ricans, while also reporting findings suggestive of disease-enriched eQTL.

## Methods

### Subject recruitment and study protocol

From March of 2009 to June of 2010, children in San Juan were chosen from randomly selected households, as previously described [4]. In brief, households in the Standard Metropolitan Area of San Juan were selected by a multistage probability sample design. Primary sampling units (PSUs) were randomly selected neighborhood clusters based on the 2000 U.S. census, and secondary sampling units were randomly selected households within each individual PSU. A household was eligible if ≥1 resident was a child 6–14 years old. A total of 6,401 households were contacted: 1,111 had ≥1 child who met inclusion criteria other than age (four Puerto Rican grandparents and residence in the same household for ≥1 year). Of these 1,111 households, 438 had ≥1 eligible child with asthma (defined as physician-diagnosed asthma and wheeze in the prior year). From these 438 households, one child with asthma was selected (at random if there was more than one such child). Similarly, only one child without asthma was randomly selected from the remaining 673 households. In order to reach a target sample size of ~700 children, we attempted to enroll 783 of the 1,111 eligible children. Parents of 105 of these 783 children refused to participate or could not be reached, leaving 678 study participants (351 children with asthma and 327 control subjects); DNA was extracted from blood samples collected in 592 children, of whom 583 had sufficient DNA for genome-wide genotyping. Total RNA was extracted from additional whole-blood samples collected in PaxGene tubes for the last 142 study participants (71 children with asthma and 71 control subjects).

Written parental consent was obtained for participating children, from whom written assent was also obtained. The study was approved by the Institutional Review Boards of the University of Puerto Rico (San Juan, Puerto Rico), Brigham and Women’s Hospital (Boston, MA), and the University of Pittsburgh (Pittsburgh, PA).

### Genome-wide genotyping

Subjects were genotyped at ~2.5 million single nucleotide polymorphisms (SNPs) using the HumanOmni2.5 BeadChip (Illumina, Inc., San Diego, CA), as previously described [4]. Subjects with a call rate <95% were removed from the analysis. SNPs were removed if they were not in Hardy–Weinberg equilibrium (P<10^-6^) in control subjects, had minor allele frequency lower than 5% or a failure rate greater than 5%. After all subject and marker quality control steps were completed, we had data on ~1.3 million SNPs from 560 subjects.

### Genome-wide expression analysis in whole blood

RNA was extracted from whole blood using PAXgene blood miRNA kits (Qiagen Inc). Globin transcripts were then depleted using GLOBINclear kit (Life technologies). RNA quality and concentration were determined using Agilent RNA 6000 Nano kit (Agilent technologies). Of the 141 children with RNA samples, 121 also had GW genotypes and are included in this analysis. GW gene expression was thus measured in 121 whole-blood globin-cleaned RNA samples at the University of Pittsburgh Genomics and Proteomics Core Laboratories, using the HumanHT-12 v4 Expression BeadChip (Illumina Inc). Background subtraction and quantile normalization were performed using the Lumi package implemented in R (version 3.0.1). Probes with > 70% absent points among all 121 samples were retained in the downstream analysis. A total of ~15,000 probes and ~1.3 million SNPs were included in the final eQTL analysis.

### Principal component analysis (PCA)

Principal component analysis was performed using SNP data and mRNA expression data separately. PCs derived using SNPs and expression data can capture different sources of variation due to global ancestry, unknown environmental exposures, or technical factors. The first two eigenvectors derived from genotypic data were included as covariates in our primary association analysis. The first ten eigenvectors derived from expression data were additionally included as covariates in our secondary association analysis (see below).

### Association analysis

A multivariate linear regression analysis was performed for each pair of mRNA expression *y*_*i*_ and SNP *x*_*j*_ for the *i* ^*th*^ transcript probe and the *j* ^*th*^ SNP. This analysis was conducted separately in subjects with and without asthma, as well as in all subjects. All multivariate models were adjusted for age, gender, and the first two principal components derived from whole-genome SNP data (to control for potential population stratification); models for the analysis including all subjects were additionally adjusted for asthma status. To maximize our statistical power, we repeated the analysis after additional adjustment for the first ten principal components derived from mRNA expression data, as follows: $y_{i}=\alpha_{0}+\alpha_{1}\times age+\alpha_{2}\times gender+\sum_{k=1}^{12}\gamma_{k}\times PC_{k}+\beta_{1}\times x_{j}+\beta_{2}\times z+\varepsilon$, where z is an individual’s asthma status.

The eQTL analysis was performed using R package MatrixeQTL [7]. Benjamini-Hochberg False Discovery Rate (FDR) was controlled at 0.01, 0.05, and 0.1. The proportion of expression variability explained (R^2^) by each tested SNP is calculated in the linear regression for each local pair. A model without z was applied similarly in the group of asthmatics and non-asthmatics, respectively.

### Annotation and relation to other diseases

eQTL loci identified in our association analysis were then compared with three existing eQTL databases. The first dataset is from an eQTL study in primary peripheral blood CD4+ lymphocytes [8]. The second database was derived from a systematic study of diverse human populations from HapMap3 samples [9]. The third database includes two datasets (MRCE and MRCA) derived from two studies of childhood asthma in Europe using a similar Illumina platform [10]. Next, we performed a pathway analysis using Ingenuity Pathway Analysis (IPA) and compared our top eQTL genes with a GWAS catalog database [11].

## Results

### eQTL mapping

**Fig 1:**
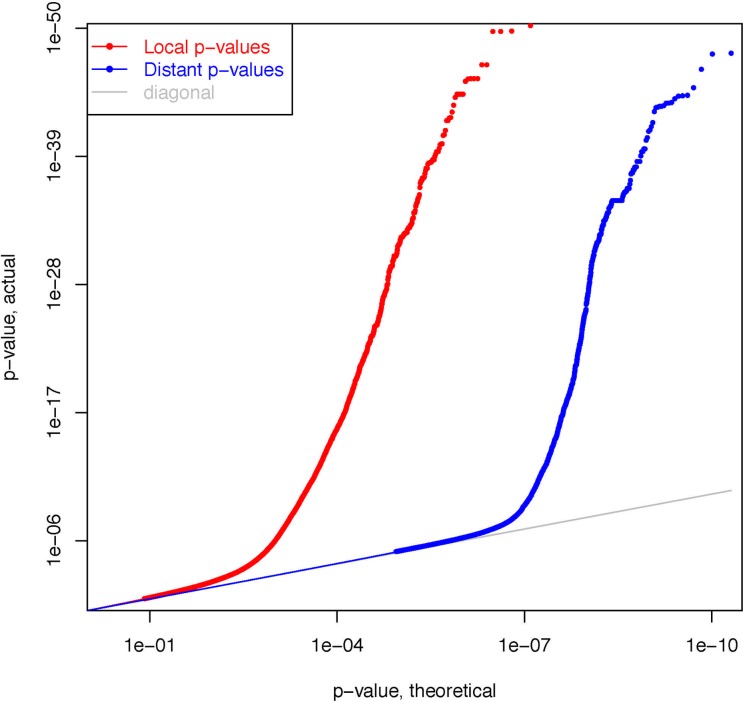
Q-Q plots of local and distal eQTLs in all samples.

We systematically examined how SNPs regulate RNA expression using 1,351,737 SNPs and 15,075 transcripts from 121 participants. We separated the mapping results by local effects and distal effects. Of a total of 20,377,435,275 tests, 12,442,690 were for local pairs and 20,364,992,585 were for distal pairs (Fig. 1). Table 1 shows the number of eQTL signals identified before and after adjustment for PCs from RNA data in all subjects, as well as the results of separate analyses in children with asthma and control subjects (both adjusted for PCs from DNA data). The majority of eQTLs were identified from local effect pairs, and we had greater statistical power to detect eQTLs after additional adjustment for PCs derived from RNA data. Adjustment for PCs resulted in the detection of an additional 22,863 local pairs under a FDR < 0.05. This finding is consistent with those from previous studies [10].

**Table 1:** Main results of eQTL mapping under different false discovery (FDR) rates.

Panel A: Without adjustment for RNA PCs, all subjects				
	Pairs	Probes	Genes	SNPs
FDR<0.01				
local	8117	921	841	7177
distal	1305	262	249	991
FDR<0.05				
local	13857	1750	1596	11922
distal	2075	507	484	1524
FDR<0.1				
local	18952	2649	2392	16087
distnat	2885	814	772	2017
Panel B: Without adjustment for RNA PCs, controls				
	Pairs	Probes	Genes	SNPs
FDR<0.01				
local	1544	277	257	1421
distal	679	151	140	443
FDR<0.05				
local	2763	508	478	2551
distal	1465	327	312	937
FDR<0.1				
local	3911	797	753	3574
distal	2455	610	584	1514
Panel C: Without adjustment for RNA PCs, cases				
	Pairs	Probes	Genes	SNPs
FDR<0.01				
local	1405	268	248	1281
distal	557	141	136	394
FDR<0.05				
local	2628	507	475	2372
distal	1170	310	297	825
FDR<0.1				
local	3837	819	761	3461
distal	1983	597	571	1353
Panel D: With adjustment for RNA PCs, all subjects				
	Pairs	Probes	Genes	SNPs
FDR<0.01				
local	22323	2242	2008	18746
distal	1391	321	306	1051
FDR<0.05				
local	36720	3808	3391	30386
distal	1851	461	446	1461
FDR<0.1				
local	50270	5461	4799	40997
distal	2272	650	627	1853

**Fig 2:**
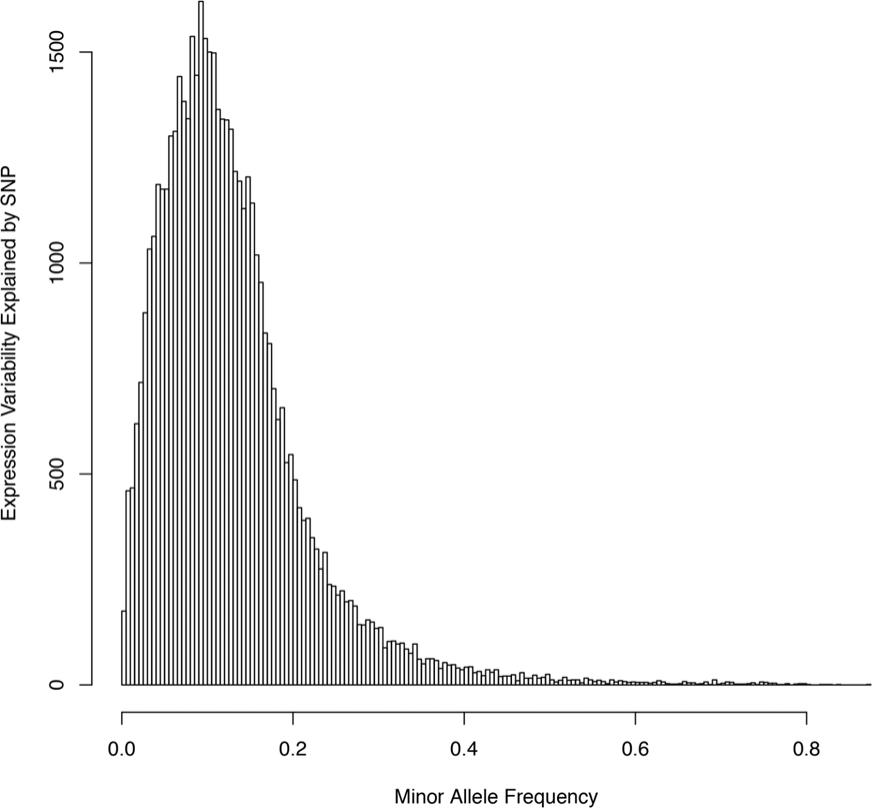
Histogram of proportion of expression variability explained.

**Fig 3:**
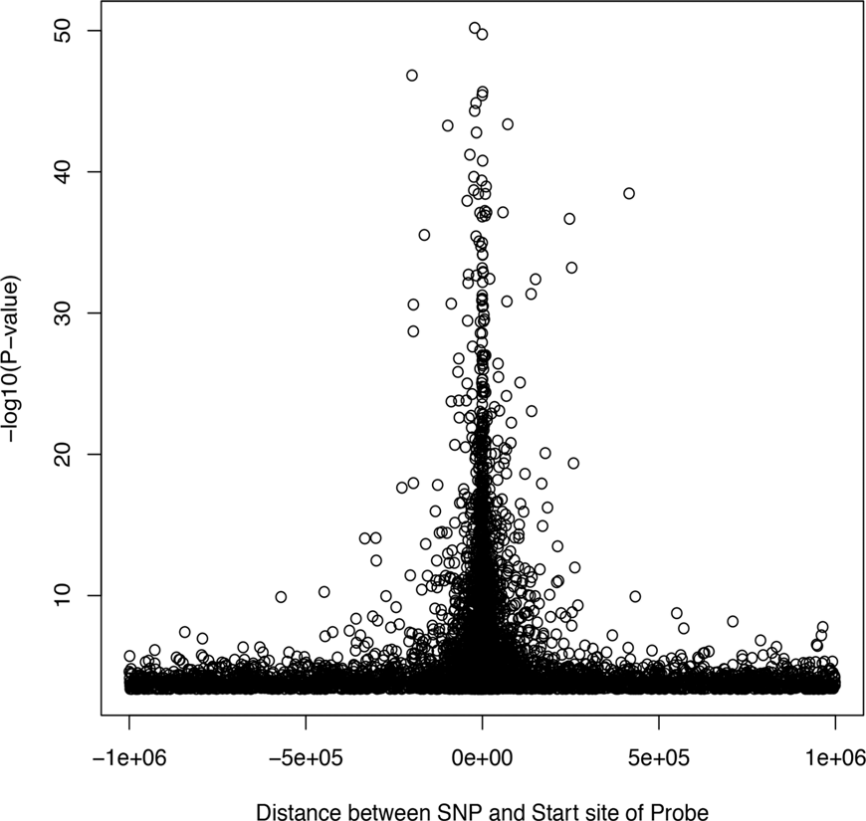
Distance to transcript start site against significance level (P value) from the eQTL association analysis.

We focused our analysis on cis-effect mapping, since these effects are the most biologically plausible and interesting. Fig. 2 is the histogram of proportion of expression variability explained for significant local effect under a FDR <0.1. The median proportion of expression variability explained was 0.11 (inter-quartile range = 0.07 to 0.16). Table 2 shows a summary of the twenty genes with the most significant associations (lowest P values). Of note, significant local eQTLs were found in a wide frequency range (S1 Fig.). There are more highly heritable local eQTLs for SNPs with higher frequency. The listed SNPs can explain between 61% and 87% of the expression variability for each gene. A complete list can be found in the supporting information (S1 Table). Fig. 3 shows a plot of the significant of each association test against to the distance between each tested SNP and its corresponding transcript. In general, SNPs tend to affect RNA expression more significantly when they are closer to the starting site for a transcript.

Even though a systematic comparison between asthmatics and non-asthmatics is limited by the sample size in each group, we list local eQTL pairs from the subgroup analysis in S2 and S3 Tables, and list case-specific and control-specific eQTLs in S4 and S5 Tables (under FDR < 0.1).

**Table 2:** Top twenty genes with most significant local associations.

SNP	Gene	CHR	A1	A2	MAF	Beta	Adjust P	R^2^
rs6877400	*SCGB3A1*	5	G	A	0.10	1.87	6.07E-44	0.871
rs2836	*RIPK5*	1	C	A	0.27	-0.69	6.07E-44	0.794
rs8458	*VAV3*	1	G	A	0.47	1.31	2.95E-41	0.757
rs14139	*IPO8*	12	A	G	0.50	0.74	2.93E-40	0.684
rs11539046	*RPL14*	3	G	A	0.29	2.01	4.21E-40	0.802
rs3773922	*TFG*	3	A	G	0.44	-0.79	1.37E-39	0.775
rs7143764	*CHURC1*	14	G	A	0.29	1.24	3.67E-39	0.705
rs10044354	*ERAP2*	5	A	G	0.35	1.15	2.91E-38	0.840
rs13290413	*LOC253039*	9	C	A	0.47	0.92	3.43E-38	0.771
rs10843881	*DDX11*	12	A	G	0.39	0.49	1.00E-37	0.822
rs10876864	*RPS26*	12	A	G	0.47	-1.10	3.03E-36	0.769
rs10088428	*HMBOX1*	8	A	G	0.28	-0.74	7.55E-36	0.733
rs6464103	*TMEM176A*	7	G	A	0.26	1.14	8.66E-35	0.714
rs323719	*CCDC23*	1	A	G	0.19	0.80	1.49E-34	0.704
rs2340518	*DPRXP4*	17	G	A	0.16	0.87	3.64E-34	0.750
rs104664	*FAM118A*	22	G	A	0.14	1.15	6.42E-34	0.796
rs4731533	*IRF5*	7	A	G	0.50	0.97	9.90E-34	0.757
rs2395943	*PEX6*	6	A	G	0.34	-0.77	1.02E-33	0.749
rs2056613	*WDR48*	3	C	A	0.43	-0.41	1.05E-33	0.613
rs1541533	*SNORD14A*	11	G	A	0.08	0.82	2.80E-33	0.660

For each gene, the most significant SNP was reported. Beta is the increase (+) or decrease (-) unit of transcript per unit increase of reference allele (A1). Reported p-value is adjusted by FDR. R^2^ is the proportion of expression variability explained by the reported SNP.

### Comparisons with existing eQTL databases

Studies reporting identification of eQTLs differ with regard to the tissues analyzed, the platforms utilized for genotyping and generation of RNA microarrays, analytical methods, and the race or ethnicity of subjects. We did focus on the identification of findings that overlap with those from previous studies. For simplicity, we focused on official gene symbols, which were then compared with those reported in other studies. It is worth noting that our limited statistical power may have led to false negative results.

In the first eQTL study we used for comparison [8], a genome-wide scan was conducted in 200 subjects for SNP genotyping and RNA expression assessment (in peripheral blood CD4+ lymphocytes). Interestingly, a few genes in Table 2 for this study (*SCGB3A1*, *IPO8*, *CHURC1*, and *FAM118A*) were among the top 34 genes with more than 50% expression variability explained in that earlier study. The second study identified eQTLs with local effects using GW genotypic data and GW expression data from Epstein-Barr virus (EBV)-transformed lymphoblastoid cell lines in 726 subjects in eight populations (109 subjects of European ancestry, 80 Han Chinese, 82 Gujarati Indians, 82 Japanese, 82 Luhya from Kenya, 45 subjects of Mexican ancestry 138 Maasai from Kenya, and Yoruba from Nigeria) in the HapMap 3 project [9]. In the third and most recent study, Liang et al. conducted eQTL mapping using GW genotypes (directly genotyped and imputed) and GW expression data from lymphoblastoid cell lines in two cohorts of European nuclear families ascertained through a child with asthma (the MRCA panel) or eczema (the MRCE panel)[10]. As shown in Table 3, a majority of the genes identified in the current study overlap with those identified in the studies conducted using HapMap3 or the MRCA/MRCE panels. The average percentage for such overlap is approximately 85% and 69% under FDR thresholds of 0.05 and 0.01, respectively. In the comparison with MRCA and MRCE datasets, we observed a fairly consistent pattern for a longer gene list. In fact, the numbers shown above are likely underestimated due to limited power [12].

**Table 3:** Comparison of eQTL loci identified in this study with those identified in other studies (HapMap, MRCA and MRCE).

HapMap				
POPULATION	Listed Gene	Overlapping Gene	FDR<0.05	FDR<0.01
CEU	660	252	215 (85.32%)	175 (69.44%)
CHB	774	300	251 (83.67%)	191 (63.67%)
GIH	701	258	221 (85.66%)	178 (68.99%)
JPT	796	297	254 (85.52%)	196 (65.99%)
LWK	774	285	246 (86.32%)	203 (71.23%)
MEX	473	159	136 (85.53%)	110 (69.18%)
MKK	950	351	304 (86.61%)	222 (63.25%)
YRI	802	260	230 (88.46%)	179 (68.85%)
MRCA	7698	2100	1593 (75.86%)	1001 (47.67%)
MRCE	8843	1662	1247 (75.03%)	785 (47.23%)

The column of “Listed Gene” is the number of genes reported in the original study. The column of “Overlapped Gene” is the number of overlapping genes between our eQTL study and compared study.

### Functional analysis and disease association

Next, we investigated if eQTL mapped genes are enriched for disease associations. We downloaded the latest version (released on April 1, 2014) of the GWAS Catalog from the NIH website. We used the "Reported Gene(s)" and "Mapped_gene" columns in the catalog file to map the genes in our study. We matched the IDs with the corresponding Disease/Trait description from the catalog, and then summarized this information in a combined table (S6 Table). It is worth noting that well-known susceptibility genes for asthma were identified in our list (S7 Table). For example, SNP rs8067378 explained nearly 24% of the variability in expression of *ORMDL3* (adjusted p-value < 10^-6^) in the current study, and has been associated with asthma in several studies [13, 14].

To identify diseases associated with our top eQTL-enriched genes, we conducted an Ingenuity Pathway Analysis on the top 500 genes. Endocrine, gastrointestinal, immunological, metabolic, and dermatological diseases appeared to be ranked at the top of the results for this analysis. Similar results were obtained when selecting a different number of top genes (e.g. 100, 300).

## Discussion

We have presented and made available our findings from an eQTL study of a well-characterized Hispanic cohort of Puerto Rican children. Our results contribute to the understanding of gene expression regulation in Puerto Ricans, while also complementing existing eQTL databases in other ethnic groups. eQTL loci identified in the current analysis will help prioritize follow up of findings from other studies of complex diseases, such as GWASs.

We recognize several limitations of our study. Firstly, we had limited statistical power because of our sample size. Thus, we were unable to detect rare or low impact eQTLs and likely underestimated the number of genes overlapping with those reported in other studies. Moreover, we cannot confidently identify eQTLs differing between Puerto Ricans and other racial/ethnic groups and between asthmatics and non-asthmatics. Subject recruitment in ongoing projects will further increase our sample size and thus our statistical power to detect additional eQTL, particularly those with modest effects. Secondly, we assessed GW expression in whole-blood, which contains a heterogeneous population of cells (e.g. subpopulations of white blood cells and platelets). However, our findings are comparable to those reported using GW expression data from CD4+ lymphocytes or lymphoblastoid cell lines. Thirdly, we limited our comparisons with previous studies to official gene symbols, largely because of complex differences across studies. An exact comparison of SNP-transcript pairs would have provided more accurate biological interpretations. Fourthly, our small sample size precluded incorporation of racial ancestry information into the analysis, which would have facilitated identification of population- or ethnic-specific eQTLs. Detection of such eQTLs is important and would contribute to gene mapping for complex diseases in racially admixed populations, such as Puerto Ricans. We plan future studies integrating genetic, epigenetic and mRNA expression data to further enhance our knowledge of the biological mechanisms underlying complex diseases in Puerto Ricans and other racially admixed populations.

## Supporting Information

S1 Fig Proportion of expression variability explained by SNP minor allele frequency. (TIFF)

S1 Table A list of significant local-effect eQTL pairs under FDR < 0.1. For each probe/gene, only the top SNP was retained.
(CSV)

S2 Table A list of top identified eQTL genes overlapping with genes associated with any trait in the GWAS catalog. (CSV)

S3 Table A list of top identified eQTL genes overlapping with genes associated with asthma-related traits in the GWAS catalog. (CSV)

S4 Table A list of significant local-effect eQTL pairs in cases under FDR < 0.1. (CSV)

S5 Table A list of significant local-effect eQTL pairs in controls under FDR < 0.1. (CSV)

S6 Table A list of significant local-effect eQTL pairs in cases but not in controls (case specific) under FDR < 0.1. (CSV)

S7 Table A list of significant local-effect eQTL pairs in controls but not in cases (control specific) under FDR < 0.1. (CSV)

## References

[1] Cookson W, Liang L, Abecasis G, Moffatt M, Lathrop M. Mapping complex disease traits with global gene expression. Nat Rev Genet. 2009;10:184–94. 10.1038/nrg2537 19223927 PMC4550035

[2] Dixon AL, Liang L, Moffatt MF, Chen W, Heath S, Wong KC, et al A genome-wide association study of global gene expression. Nat Genet. 2007;39:1202–7. 17873877 10.1038/ng2109

[3] Rockman MV, Kruglyak L. Genetics of global gene expression. Nat Rev Genet. 2006;7:862–72. 17047685 10.1038/nrg1964

[4] Brehm JM, Acosta-Perez E, Klei L, Roeder K, Barmada MM, Boutaoui N, et al African ancestry and lung function in Puerto Rican children. J Allergy Clin Immunol. 2012;129:1484–90 e6. 10.1016/j.jaci.2012.03.035 22560959 PMC3367038

[5] Rosas-Salazar C, Ramratnam SK, Brehm JM, Han YY, Boutaoui N, Forno E, et al Prematurity, atopy, and childhood asthma in Puerto Ricans. J Allergy Clin Immunol. 2014;133:357–62. 10.1016/j.jaci.2013.09.003 24139607 PMC3960360

[6] Forno E, Acosta-Perez E, Brehm JM, Han YY, Alvarez M, Colon-Semidey A, et al Obesity and adiposity indicators, asthma, and atopy in Puerto Rican children. J Allergy Clin Immunol. 2014;133:1308–14, 14 e1–5. 10.1016/j.jaci.2013.09.041 24290290 PMC4013276

[7] Shabalin AA. Matrix eQTL: ultra fast eQTL analysis via large matrix operations. Bioinformatics. 2012;28:1353–8. 10.1093/bioinformatics/bts163 22492648 PMC3348564

[8] Murphy A, Chu JH, Xu M, Carey VJ, Lazarus R, Liu A, et al Mapping of numerous disease-associated expression polymorphisms in primary peripheral blood CD4+ lymphocytes. Hum Mol Genet. 2010;19:4745–57. 10.1093/hmg/ddq392 20833654 PMC2972694

[9] Stranger BE, Montgomery SB, Dimas AS, Parts L, Stegle O, Ingle CE, et al Patterns of cis regulatory variation in diverse human populations. PLoS Genet. 2012;8:e1002639 10.1371/journal.pgen.1002639 22532805 PMC3330104

[10] Liang L, Morar N, Dixon AL, Lathrop GM, Abecasis GR, Moffatt MF, et al A cross-platform analysis of 14,177 expression quantitative trait loci derived from lymphoblastoid cell lines. Genome Res. 2013. 10.1101/gr.142521.112 PMC3613588 23345460

[11] Welter D, MacArthur J, Morales J, Burdett T, Hall P, Junkins H, et al The NHGRI GWAS Catalog, a curated resource of SNP-trait associations. Nucleic Acids Res. 2014;42:D1001–6. 10.1093/nar/gkt1229 24316577 PMC3965119

[12] Ding J, Gudjonsson JE, Liang L, Stuart PE, Li Y, Chen W, et al Gene expression in skin and lymphoblastoid cells: Refined statistical method reveals extensive overlap in cis-eQTL signals. Am J Hum Genet. 2010;87:779–89. 10.1016/j.ajhg.2010.10.024 21129726 PMC2997368

[13] Moffatt MF, Kabesch M, Liang L, Dixon AL, Strachan D, Heath S, et al Genetic variants regulating ORMDL3 expression contribute to the risk of childhood asthma. Nature. 2007;448:470–3. 17611496 10.1038/nature06014

[14] Hancock DB, Romieu I, Shi M, Sienra-Monge JJ, Wu H, Chiu GY, et al Genome-wide association study implicates chromosome 9q21.31 as a susceptibility locus for asthma in mexican children. PLoS Genet. 2009;5:e1000623 10.1371/journal.pgen.1000623 19714205 PMC2722731

