# Supplementary figures and images for: Expression Quantitative Trait Loci (eQTL) Mapping in Puerto Rican Children
Source: PLoS One. 2015 Mar 27;10(3):e0122464. doi: 10.1371/journal.pone.0122464 (PMC4376710; doi:10.1371/journal.pone.0122464)

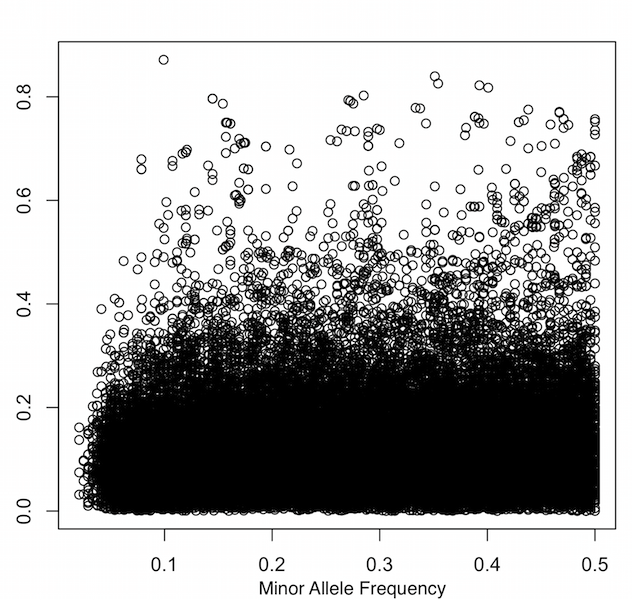

Supplement: S1 Fig — (TIFF) [file pone.0122464.s001.tiff]
